# The Impact of Laparoscopic Myomectomy on Pregnancy Outcomes: A Systematic Review

**DOI:** 10.3390/jpm14040340

**Published:** 2024-03-25

**Authors:** Ligia Balulescu, Simona Brasoveanu, Marilena Pirtea, Dorin Grigoras, Cristina Secoșan, Flavius Olaru, Dragos Erdelean, Mădălin-Marius Margan, Alexandru Alexandru, Cristiana-Smaranda Ivan, Laurențiu Pirtea

**Affiliations:** 1 Department of Obstetrics and Gynecology, Victor Babes University of Medicine and Pharmacy, 300041 Timisoara, Romania; ligia.balulescu@umft.ro (L.B.); marilena.pirtea@umft.ro (M.P.); grigoras.dorin@umft.ro (D.G.); secosan.cristina@umft.ro (C.S.); olaru.flavius@umft.ro (F.O.); erdelean.dragos@umft.ro (D.E.); pirtea.laurentiu@umft.ro (L.P.); 2 Department of Functional Sciences, Discipline of Public Health, Victor Babes University of Medicine and Pharmacy, 300041 Timisoara, Romania; margan.madalin@umft.ro; 3 Department of general medicine, Victor Babes University of Medicine and Pharmacy, 300041 Timisoara, Romania; alexandru.alexandru@student.umft.ro (A.A.); smaranda.ivan@student.umft.ro (C.-S.I.)

**Keywords:** pregnancy, leiomyoma, myomectomy, fertility, laparoscopy, robot-assisted laparoscopic myomectomy

## Abstract

Study objective: The objective of this systematic review is to investigate the impact of laparoscopic myomectomy techniques on pregnancy outcomes, with a specific focus on the correlation between the type of suture used during the procedure and the incidence of uterine rupture. Additionally, the study aims to examine how the localization and size of myomas, key factors in laparoscopic myomectomy, may influence fertility outcomes. Data Sources: extensive searches were conducted using MDPI, PubMed, Web of Science, and Cochrane Library databases from 2008 to November 2023. Methods of Study Selection: The study involved women of reproductive age diagnosed with fibroids who underwent surgical removal of fibroids using either laparotomy or laparoscopy. The evaluation of pregnancy outcomes focused on indicators such as live birth rates, miscarriage rates, stillbirth rates, premature delivery rates, and cases of uterine rupture. Quality assessment was systematically performed by employing the National Institutes of Health Study Quality Assessment Tools, with the subsequent formulation of clinical recommendations that were meticulously graded in accordance with the robustness of the underlying evidence. Results: The pregnancy outcomes post-myoma treatment, as reflected in one of the presented tables, show a promising number of pregnancies and live births, but also indicate the potential risks of miscarriages and preterm births. The diversity in outcomes observed among various studies underscores the imperative for tailored patient care, as well as the necessity for additional research aimed at optimizing fertility and pregnancy outcomes following myoma treatment. Conclusion: This study offers insights into the criteria for patient selection and intraoperative methodologies specifically related to laparoscopic myomectomy. To enhance our understanding of the associations between fibroid characteristics (location, size) and reproductive outcomes, additional research is warranted, particularly through well-designed clinical trials.

## 1. Introduction

The primary aim of this study is to conduct a systematic literature review with the purpose of delineating optimal practices for laparoscopic myomectomy in women with fibroids who are actively seeking to enhance their fertility. Key areas of scrutiny encompass factors related to conception or post-myomectomy pregnancy outcomes; the fertility repercussions of preoperative findings related to laparoscopic myomectomy; and the impact of surgical techniques on subsequent reproductive outcomes.

Leiomyomas, commonly known as uterine fibroids, represent a prevalent and clinically significant pathology within the gynecological domain, impacting approximately 70% of the female population before menopause [1]. These benign myometrial neoplasms are implicated in a range of clinical presentations, including menorrhagia, dysmenorrhea, and increased urinary frequency, thereby contributing to a compromised quality of life. Their substantial role in gynecologic morbidity is underscored by their position as the primary indication for hysterectomies in the United States, accounting for nearly 40% of these procedures. Population-based assessments have revealed a high frequency of fibroids among asymptomatic premenopausal women, indicating a broader epidemiological impact than symptom-driven data might suggest. Disproportionate disease expression has been observed among African American women, characterized by heightened incidence, precocious onset, and intensified symptomatology.

The intricate interrelationship between fibroid pathology and female fertility presents a substantial clinical challenge. Research has established a clear association between the presence of fibroids and infertility. However, the therapeutic approach to optimizing fertility in affected women remains a subject of ongoing debate. Current evidence supports the role of hysteroscopic myomectomy in improving fertility, as endorsed by the American Society of Reproductive Medicine (ASRM). Additionally, the ASRM recommends considering non-hysteroscopic myomectomy for intramural fibroids with a submucosal component, aiming to enhance reproductive potential [2]. Nevertheless, the absence of definitive criteria outlining the extent of uterine cavity distortion caused by fibroids remains a substantial clinical challenge.

Certainly, infertility can arise from various factors, including ovulation disorders, such as polycystic ovary syndrome; advanced maternal age; endometriosis; pelvic adhesions; lifestyle factors; genetic factors; uterine anomalies [3]; tubal factors; or abnormalities in the uterus or cervix (fibroids or polyps).

Uterine myomas, characterized by their variability in size, location, and number, are implicated in infertility through diverse mechanisms. Distortion of local anatomy, including the endometrial cavity and tubal ostia, along with alterations in the uterine contour, can hinder the movement of gametes and embryos. Functional changes such as increased uterine contractility and chronic inflammation disrupt normal reproductive processes, leading to decreased pregnancy rates. Endocrine imbalances within the uterus may also contribute to infertility. Paracrine effects on adjacent endometrium, alterations in cytokine levels (glycodelin—a progesterone-regulated glycoprotein—and interleukin 10 levels decrease), and disruptions in the endo-myometrial junctional zone (reduction of macrophages and the concentration of uterine natural killer cells) further complicate fertility. Reduced endometrial receptivity, evidenced by the lowered expression of genes essential for implantation (HOXA10 and HOXA11 mRNA), presents another facet of myoma-related infertility. Additionally, myomas may impact sexual function, leading to pelvic pain, dyspareunia, and decreased libido, potentially reducing the frequency of sexual intercourse and natural conception opportunities. Understanding these multifaceted mechanisms is crucial for managing infertility associated with uterine myomas [4].

The surgical management of fibroids, particularly concerning reproductive outcomes, requires more nuanced guidance. Despite the fact that the effectiveness of non-hysteroscopic myomectomy is recognized in the field of fibroid management, there is a lack of comprehensive directives on the intricacies of such procedures, whether performed via laparotomy or minimally invasive techniques.

The differences in pregnancy risks between conservative approaches and surgical myomectomy for fibroid treatment remain uncertain. Through a comprehensive analysis of the existing literature, we intend to provide insights into the nuanced relationships between surgical techniques, suture types, myoma characteristics, and their collective influence on the reproductive outcomes of women undergoing laparoscopic myomectomy.

## 2. Materials and Methods

### 2.1. Search Strategy

The PRISMA (Preferred Reporting Items for Systematic Reviews and Meta-Analyses) methodology guided this study [5]. Extensive searches were conducted through MDPI, PubMed, Web of Science, Science Direct, and Cochrane Library databases using specific keywords, including myomectomy, laparoscopy, leiomyoma, leiomyomata, pregnancy, infertility, and pregnancy loss. The inclusive scope of reviewed articles comprised primary research, encompassing randomized controlled trials, cohort studies, prospective and retrospective studies, case–control studies, case reports, and case series, spanning from the year 2008 to November 2023. Additional studies were sought in the references of identified publications, including prior narrative reviews and meta-analyses [5].

### 2.2. Study Selection

This study exclusively considered full-length articles. The evaluation of pregnancy outcomes focused on indicators such as live birth rates, miscarriage rates, stillbirth rates, premature delivery rates, and cases of uterine rupture. Exclusion criteria included articles involving patients who did not undergo abdominal myomectomy, specifically through a laparoscopic approach. Additionally, papers reporting on surgical interventions other than a laparoscopic myomectomy without a direct comparison to patients undergoing a laparoscopic myomectomy were excluded, as well as studies lacking data on fertility outcomes.

### 2.3. Data Extraction

**Figure 1:**
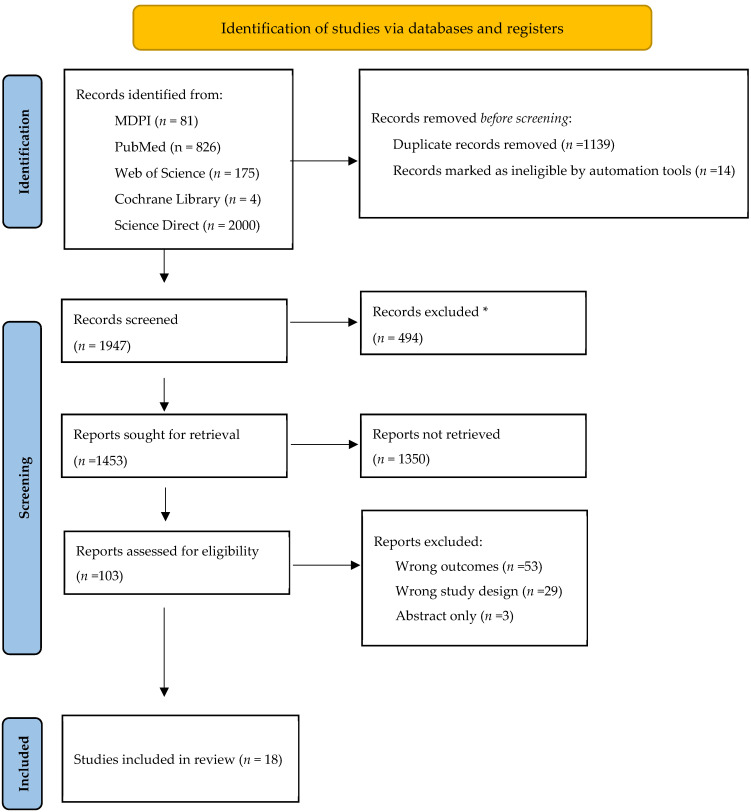
Flowchart of the selection process. * did not address the subjects of this review.

Following identification, all located publications underwent independent review by two evaluators (SI and AA). Data extraction was carried out independently using a standardized form. Information was compiled into tables, detailing authors’ names, year of publication, study design, control, intervention groups, mean age, and surgical findings. Fertility outcomes, as reported in the study, varied by topic, and encompassed number of pregnancies; miscarriages and live birth rates; mode of delivery; time taken to conceive; and type of suture. Any discrepancies were resolved through consensus. The selection process, adhering to PRISMA guidelines, is illustrated in Figure 1.

### 2.4. Quality Assessment

Two reviewers conducted a quality assessment utilizing the National Institutes of Health Study Quality Assessment Tools, accessible at www.nhlbi.nih.gov/health-topics/study-quality-assessment-tools (see Table 1, accessed on 18 December 2023).

## 3. Results

### 3.1. Study Selection

Upon the initial search, 3086 studies were identified (see Figure 1). After screening article titles and abstracts, 103 underwent a full-text review, resulting in the removal of 85 studies due to various reasons, including outcomes *(n* = 53), study design (*n* = 29), and lack of full-text publication (*n* = 3). In total, 18 studies were included in this systematic review for qualitative analysis, and the findings are detailed below.

### 3.2. Study Demographics

The mean age of women across these studies ranged broadly from around 30 to 40 years, with most studies reporting a mean age in the early to mid-thirties, signifying a crucial overlap with both the peak reproductive years and the prevalent age range for myoma development (Table 2). For instance, Yu-cui Tian’s study in 2014 [13] had an average age of 30.28 ± 3.99, while Yeon Hee Hong’s 2021 study [22] reported a higher average age of 40.6 ± 6.6. This variation in age is crucial as it potentially impacts fertility outcomes post-myomectomy.

A discernible disparity in sample sizes was evident across the studies, encompassing more diminutive cohorts, such as Fukuda [18] with 48 participants, and substantially larger groups, as exemplified by the study by Kumakiri [17], which incorporated 1334 women. This disparity in sample sizes is significant as it may affect the generalizability and reliability of the study findings, and thus influence the interpretability of the aggregate data in the context of broader population-level implications.

The studies exhibited diversity in the number of myomas per patient, with certain individuals manifesting a solitary myoma, while others presented with multiple myomas. This heterogeneity across myoma counts is of clinical significance as the number could influence the complexity of the surgical procedure, the postoperative recovery, and potentially the subsequent pregnancy outcomes.

The operative technique primarily employed across these studies is laparoscopic myomectomy (LSM), with some variations like robot-assisted laparoscopic myomectomy (RALSM) and laparoscopic assisted intracapsular myoma (LAIM). These techniques reflect the evolving nature of surgical interventions in treating myomas.

The number of myomas treated exhibited variability, as certain studies reported an average of a single myoma, while others documented the treatment of multiple myomas. This diversity indicates that laparoscopic techniques are employed in a wide range of clinical scenarios, from simple to complex myoma presentations.

The data collated from these studies encompass a spectrum of pregnancy outcomes, including instances of live births, miscarriages, and preterm deliveries. The explicit categorization of ‘Preterm’ deliveries and the ‘Mode of Delivery’ (cesarean or vaginal) within the data highlights these as critical outcome variables. Furthermore, the variability in pregnancy outcomes and their frequency across different studies suggests disparities in fertility rates and pregnancy-related complications among the different cohorts, indicative of the multifactorial nature of reproductive outcomes post-myoma treatment.

Pregnancy occurrences documented in the studies indicate that women achieved conception subsequent to myoma treatment. For instance, in the study by Yu-Jin Koo [9], there were 523 pregnancies, indicating a high rate of conception post-treatment.

The number of live births is a critical measure of successful pregnancy outcomes. For example, in the same study by Yu-Jin Koo [9], out of 523 pregnancies, there were 401 live births, showcasing a substantial success rate.

Miscarriages are an important consideration when evaluating the outcomes of pregnancies post-myoma treatment. The studies show varying rates of miscarriages. For example, Tina Sybille Bernardi [8] reported 13 miscarriages out of 55 pregnancies, which is a notable figure and might suggest a potential impact of myomas or their treatment on pregnancy viability.

In contrast, Myo Sun Kim [6] reported a lower number of miscarriages (3 out of 54 pregnancies), indicating variability across different cohorts and possibly different treatment protocols or myoma characteristics.

Preterm delivery is a concern in pregnancies post-myoma treatment. For instance, in the study by Tina Sybille Bernardi [8], there were 3 preterm deliveries out of 55 pregnancies.

However, some studies, like those of Prapas [10] and Ordás [15], did not provide data on preterm deliveries (marked as NA), highlighting a gap in comprehensive outcome reporting.

The mode of delivery, whether vaginal or cesarean, is an important aspect of pregnancy outcomes. For example, Yu-Jin Koo [9] reported 350 cases of cesarean delivery out of 523 pregnancies, suggesting a higher preference or need for cesarean sections in this cohort.

The reasons behind the choice of delivery mode can be multifactorial, including the history of myoma treatment, the location and size of any remaining myomas, and other obstetric considerations.

There is considerable variability in the reported outcomes across different studies, which might be attributed to factors like the patient’s age, the characteristics of the myomas, surgical techniques, and individual patient health.

Some studies have missing data (marked as NA), which limits the ability to draw comprehensive conclusions about pregnancy outcomes post-myoma treatment across a broader population.

In conclusion, the pregnancy outcomes post-myoma treatment, as reflected in the table below, show a promising number of pregnancies and live births, but also indicate the potential risks of miscarriages and preterm births. The variability in these outcomes across different studies highlights the need for individualized patient care and further research to optimize fertility and pregnancy results post-myoma treatment.

### 3.3. SPLSM vs. LSM

In a case–control study involving 135 patients, pregnancies were investigated following two different surgical approaches for patients with fibroids smaller than 8 cm in diameter and with a distance of less than 5 mm between the fibroid and the serosa, as seen on ultrasound imaging. The study’s findings indicated that there were no significant differences between the two groups with respect to various factors, including patient demographics, fibroid characteristics, and fertility outcomes. These fertility outcomes encompassed pregnancy rates (50% vs. 67%, *p* = 0.38), term delivery rates (58% vs. 67%, *p* = 0.38), rates of vaginal delivery (43% vs. 0%, *p* = 0.40), and rates of miscarriage (25% vs. 11%, *p* = 0.38) [23].

Within a retrospective cohort study comprising a total of 502 patients, 376 of them (74.9%) fell within the reproductive age bracket. Within the group of patients of reproductive age, 56 individuals expressed a strong desire to have children following their surgical procedures. Among these 56 patients, 42 achieved successful pregnancies, resulting in 39 live births. Additionally, two pregnancies (3.6%) ended in miscarriages, and one patient was lost to follow-up once her pregnancy was confirmed. Among these live births, 36 (92.3%) were term deliveries with an average gestational age of 38.2 ± 0.9 weeks, while the remaining 3 (7.7%) were premature deliveries, with an average gestational age of 34.0 ± 3.5 weeks. Cesarean section was the predominant mode of delivery, accounting for 36 infants (92.3%), although 3 infants (7.7%) were delivered vaginally [22].

### 3.4. Prevalence and Characteristics of Myomas

Regarding the myoma location, the studies highlighted various locations for myomas, including intramural, submucosal, subserosal, interligamentary, and cervical positions (Table 3). Bernardi’s study [8] reported a significant prevalence of partially intramural, intramural with endometrium contact, and subserous fibroids. In contrast, Pepin [12] and Paul P. G. [11] found a higher occurrence of intramural myomas, while Guangping Wu’s study [16] identified a combination of intramural and submucosal myomas as common.

The average number of myomas per woman varied slightly across studies, with Bernardi, Pepin [12], and Paul P. G. [11] reporting an average of 2 myomas per woman, while Guangping Wu’s study [16] observed a higher average of 3.9 myomas.

In these studies, the proportion of pregnant women was noteworthy, with Guang-ping Wu’s study [16] reporting a higher number of pregnant women (253 out of 224) compared to the total number of women studied. This warrants a significant interest in the implications of myoma treatment on fertility and pregnancy outcomes. The diversity in myoma locations and the numbers of myomas underscore the need for personalized treatment approaches.

### 3.5. Intraoperative Techniques (Type of Suture) and Uterine Rupture

Upon reviewing information from various retrospective cohort studies that included data regarding uterine rupture, several conclusions emerged (Table 4). The use of multilayer sutures, as investigated by Myo Sun Kim [6] and Fukuda [18], seems associated with promising outcomes, with the latter reporting no instances of uterine rupture among 48 participants. Conversely, studies by Tina Sybille Bernardi [8] and Yu-Jin Koo [9] employing intracorporeal techniques reveal a higher incidence of uterine rupture, suggesting a potential correlation between this approach and increased risk. Interestingly, Alessandro Arena’s [14] investigation into barbed and non-barbed techniques demonstrates no uterine ruptures among 83 and 81 participants, respectively, indicating a possible lower risk associated with these methods. Furthermore, Paul P. G.’s [11] study corroborates these findings, reporting no uterine ruptures in cohorts employing both barbed and non-barbed techniques. This synthesis of evidence underscores the significance of suture selection in mitigating the risk of uterine rupture during fertility-related procedures, with potential implications for clinical decision-making in reproductive health interventions.

### 3.6. Number of Myomas and Fertility Outcomes

The association between pregnancy outcomes and the quantity of myomas, also referred to as uterine fibroids, has garnered attention in multiple research studies, as delineated in Table 5 Scrutiny of the data from these investigations reveals noteworthy patterns. In general, an upward trend in the number of myomas appears to be associated with a decline in both pregnancy occurrences and successful live births, coupled with an increased incidence of preterm deliveries and miscarriages. For instance, the research conducted by Myo Sun Kim [6] and Ordás [15] demonstrates a reduction in pregnancy instances and live births as the number of myomas rises, along with an elevated miscarriage rate. Conversely, studies like Guangping Wu [16] and Kumakiri [17] suggest an adverse influence on pregnancy outcomes with a greater number of myomas, indicating a higher frequency of preterm births and delivery-related complications. Nevertheless, it is crucial to acknowledge that each study features its distinct patient demographics and methodologies, which can impact these findings. To establish a definitive correlation between myoma quantity and pregnancy outcomes, additional research and a more extensive analysis would be necessary.

## 4. Discussion

Uterine fibroids are prevalent among women of reproductive age, and myomectomy is a viable option for managing the associated symptoms among those who wish to conceive. Laparoscopic myomectomy is the preferred method due to its advantages over alternatives, including ulipristal acetate, uterine artery embolization, and fibroid thermal ablation, in improving pregnancy outcomes [24,25,26].

Therefore, a comprehensive understanding of its impact is essential for designing appropriate treatment plans. Previous research has primarily focused on the short-term and surgical outcomes of myomectomy, including blood loss and perioperative complications, with limited discussion on its effects on ovarian reserves.

The findings from various studies on post-myomectomy fertility reveal a nuanced landscape, presenting diverse demographics and outcomes. The heterogeneity in age among participants is a critical factor, spanning from the early 30s to early 40s. This age range is significant as it encapsulates both the peak reproductive years and the common age range for myoma development. The variations in age may have implications for fertility outcomes post-myomectomy, as demonstrated by the wide age range of the study participants, impacting the generalizability of the findings.

Another noteworthy aspect is the substantial variation in sample sizes observed across the studies. Ranging from smaller cohorts to significantly larger groups, the sample size differences introduce challenges in interpreting and generalizing the results. Larger cohorts, such as Kumakiri’s study with 1334 women, may provide more statistically robust findings, but may not fully capture the diversity of clinical scenarios. This variance in sample sizes underscores the need for cautious interpretation and emphasizes the importance of considering the potential impact on generalizability [17].

The heterogeneity in the number of myomas per patient is of clinical significance. It introduces variability in the complexity of the surgical procedure, postoperative recovery, and potential pregnancy outcomes. The majority of studies employed laparoscopic myomectomy (LSM), showcasing the evolving nature of surgical interventions. The range of myoma presentations, from single to multiple, further illustrates the adaptability of laparoscopic techniques to various clinical scenarios.

The spectrum of pregnancy outcomes, including live births, miscarriages, and preterm deliveries, adds complexity to the overall picture. The high number of pregnancies reported across studies, such as Yu-Jin Koo’s study with 523 pregnancies, indicates that women are able to conceive following myoma treatment [9]. However, the variability in pregnancy outcomes, particularly in rates of miscarriage and preterm delivery, suggests multifactorial influences, potentially related to myoma characteristics, treatment protocols, or patient-specific factors.

Live births, a critical measure of successful pregnancy outcomes, vary across studies. While Yu-Jin Koo’s study reported a substantial success rate with 401 live births out of 523 pregnancies [9], other studies, like Alessandro Arena’s 2021 study [14], showed higher rates of miscarriages. The discrepancies highlight the need for a nuanced understanding of factors influencing successful pregnancies post-myomectomy.

Miscarriage rates also varied among studies, emphasizing the potential impact of myomas or their treatment on pregnancy viability. Preterm deliveries—a concern in pregnancies post-myoma treatment—exhibited variability, with some studies not providing data on this outcome.

In a recent retrospective study conducted by Jeldu M., the pregnancy rate after myomectomy was 52.2% [26]. Myomectomy has been associated with a decrease in abortion rates, dropping from 43% to 24% postoperatively [8]. Other studies have also reported similar reductions in abortion rates post-myomectomy, ranging from 41–60% to 19–24% [27,28]. However, even with these improvements, the abortion rate post-myomectomy remains higher than in the general population (10–15%) [11]. The incidence of ectopic pregnancies after myomectomy was slightly higher (4%) than in the general population. This may be attributed to an overall elevated frequency of ectopic pregnancies in women with infertility [29].

Another important aspect to be discussed is the possibility of practicing robot-assisted laparoscopic myomectomy, as seen in the retrospective cohort study by Huberlant which reported that over half of the patients became pregnant after robot-assisted laparoscopic myomectomy (52.8%), with a live birth rate of 41.5% [7]. This retrospective cohort study highlights the promising role of robot-assisted laparoscopic myomectomy in achieving high pregnancy rates and live birth rates, and minimizing myomectomy-related complications. As technology continues to advance, the integration of robotic surgery in the field of gynecology holds great potential for improving patient outcomes and shaping the future of myoma management.

The mode of delivery, whether cesarean or vaginal, adds another layer of complexity to the discussion. The reported instances of cesarean deliveries, as seen in Yu-Jin Koo’s study (350 out of 523 pregnancies), raise questions about the factors influencing the choice of delivery mode post-myomectomy [9]. The absence of data on preterm deliveries in some studies, such as Prapas’s and Ordás’s, indicates a gap in comprehensive outcome reporting [10,15]. Understanding the reasons behind the choice of delivery mode and addressing missing data is crucial for a more comprehensive evaluation of post-myomectomy pregnancy outcomes.

There is a divergence of opinions among experts regarding the necessity of cesarean sections after myomectomy. Some argue that any previous uterine surgery, including myomectomy—especially after the removal of large or numerous myomas—is an indication for a cesarean section [30,31], while others believe that a cesarean section is not always necessary. Bernardi et al. reported a higher rate of cesarean sections (67%). This rate is notably higher than the median cesarean section rate (27.5%) observed in the department during the study period [8].

Uterine rupture is a rare but serious complication that can have severe consequences for both the mother and the baby. It seems that peripartum uterine rupture following laparoscopic myomectomy has been reported in the literature [32,33,34,35,36]. It has highlighted various outcomes, including nonviable fetuses mid-trimester, neonatal deaths, and instances of placental accidents such as placental abruption or percreta. The majority of uterine ruptures reportedly occurred before the onset of labor, typically in the second trimester or early third trimester. Pistofidis reported uterine rupture during pregnancy in six cases between 24–35 weeks and one case at 28 weeks during labor, with neonatal death in one twin pregnancy. 

Additional reported cases of peripartum uterine rupture following laparoscopic myomectomy were identified in the literature. Among these cases, four resulted in nonviable fetuses mid-trimester, two concluded with neonatal death, and three reported incidents of placental accidents, such as placental abruption or percreta [32,33,34,35,36].

The ideal time interval between myomectomy and pregnancy remains uncertain, lacking specific guidelines. A systematic review aimed to investigate the time lapse from myomectomy to pregnancy and assess the incidence of uterine rupture following myomectomy. The mean time appears to be 17.6 months. The shortest time from myomectomy to pregnancy was a mean of 4.3 months, with a uterine rupture occurrence rate of 0.5% occurring at a mean gestational age of 31 weeks [37]. No linear relationship was observed between the gestational age at the event and the time interval from myomectomy to conception. The available data are insufficient to recommend a minimum time interval between myomectomy and conception [37]. The same idea is supported by Koo, who found that the time between surgery and conception varied by up to 8 years and most ruptures, the number of which was very low, occurred without labor [9].

When analyzing the relationship between number of fibroids during myomectomy and the pregnancy outcome, Shue suggested that women with more than six fibroids removed were less likely to become pregnant compared to women with fewer than six fibroids removed [38]. Moreover, the increasing number of fibroids removed is associated with the risk of intraoperative complications, such as significant intraoperative blood loss, which can be controlled by using a peri-cervical tourniquet, or temporary occlusion of the hypogastric artery in reducing blood loss during laparoscopic myomectomy [39,40].

The earlier findings propose that risk factors for uterine rupture include the single-layered closure of the uterine wall and the frequent application of electrocautery. Specifically, research indicates that single-layer suturing on a myometrial defect increases the risk of rupture during labor by four times compared to a two-layer closure. An Italian study found that some risk factors can predict uterine rupture in pregnancy—myoma size (>5 cm), number (>3), and type (intraligamentous). In line with expert viewpoints, a cesarean section is advised when over 50% of the myometrium is compromised, as it is the myometrium, not the endometrium, that plays a crucial role in maintaining uterine integrity [41].

### 4.1. Variability in Reported Outcomes and Missing Data

The considerable variability in reported outcomes across studies may be attributed to factors like patient age, myoma characteristics, surgical techniques, and individual patient health. The presence of missing data in some studies, marked as NA, limits the ability to draw comprehensive conclusions about pregnancy outcomes post-myoma treatment across a broader population. This gap emphasizes the importance of standardized reporting and thorough data collection in enhancing the reliability and applicability of findings.

### 4.2. Overall Variability and Limitations

The substantial variability in reported outcomes across studies can be attributed to factors such as patient age, myoma characteristics, surgical techniques employed, and the individual health statuses of patients. However, the presence of missing data in some studies poses a limitation, impeding the ability to draw comprehensive conclusions about pregnancy outcomes post-myoma treatment across diverse populations. The need for standardized reporting and more extensive data collection to enhance the reliability and applicability of findings is apparent.

## 5. Conclusions and Future Directions

The management of fibroids in women who desire to preserve their fertility remains a challenge.

Laparoscopic myomectomy is a thoroughly validated technique with well-established indications. This procedure has been shown to improve pregnancy outcomes, with an increase in pregnancy rates from 34% to 68% postoperatively. Cesarean sections may be advantageous, especially after the removal of large or numerous myomas, particularly when the endometrial cavity has been breached during myomectomy.

In conclusion, the collective findings underscore the complexity of post-myomectomy fertility outcomes. While the data show a promising number of pregnancies and live births, the variability in outcomes, including miscarriages and preterm births, highlights the necessity for individualized patient care. Future research should address missing data, standardize outcome reporting, and delve deeper into the factors influencing fertility post-myoma treatment. This multifaceted approach will contribute to a more nuanced understanding of the impact of myomectomy on fertility, guiding clinicians in optimizing patient care and providing a foundation for future advancements in this field.

## Figures and Tables

**Table 1:** Quality Assessment of the selected studies.

Study	Study Design	1	2	3	4	5	6	7	8	9	10	11	12	13	14	Quality
Myo Sun Kim [6]	Cohort	Y	Y	Y	Y	Y	N	Y	Y	Y	N	Y	N	Y	N	Good
Stéphanie Huberlant [7]	Cohort	Y	Y	Y	Y	Y	N	Y	Y	Y	N	Y	Y	Y	Y	Good
Tina Sybille Bernardi [8]	Cohort	Y	Y	Y	N	N	Y	N	N	N	N	N	NA	-	-	Poor
Yu-Jin Koo [9]	Cohort	Y	Y	Y	Y	Y	N	Y	Y	Y	N	Y	N	Y	N	Good
Prapas [10]	Cohort	Y	Y	Y	Y	Y	Y	Y	NA	NA	NA	Y	Y	Y	Y	Good
Paul P. G. [11]	Cohort	Y	Y	Y	Y	Y	Y	Y	NA	Y	Y	Y	NA	Y	Y	Good
Pepin [12]	Cohort	Y	Y	Y	Y	Y	Y	Y	NA	Y	NA	Y	Y	Y	Y	Good
Yu-cui Tian [13]	Cohort	Y	N	Y	Y	Y	Y	Y	Y	Y	NA	Y	Y	Y	Y	Good
Alessandro Arena [14]	Cohort	Y	Y	Y	Y	Y	Y	Y	NA	Y	NA	Y	NA	Y	Y	Good
Ordás [15]	Cohort	Y	Y	Y	Y	Y	Y	Y	NA	Y	NA	Y	Y	Y	Y	Good
Guangping Wu [16]	Cohort	Y	Y	Y	Y	Y	Y	Y	NA	Y	NA	Y	NA	Y	Y	Good
Kumakiri [17]	Cohort	Y	Y	Y	Y	N	Y	Y	NA	Y	NA	Y	N	Y	N	Fair
Fukuda [18]	Cohort	Y	Y	CD	CD	N	Y	Y	NA	Y	NA	Y	N	CD	N	Fair
Rebecca Flyckt [19]	Cohort	Y	Y	N	Y	N	Y	Y	Y	Y	NA	Y	N	Y	N	Fair
Pitter [20]	Cohort	Y	Y	Y	Y	N	Y	Y	Y	Y	NA	Y	N	CD	Y	Fair
Cela [21]	Cohort	Y	Y	Y	Y	Y	Y	Y	Y	Y	NA	Y	Y	Y	Y	Good
Yeon Hee Hong [22]	Cohort	Y	Y	Y	Y	Y	Y	Y	Y	Y	NA	Y	Y	Y	Y	Good
Ji Ye Kim [23]	Case–control	Y	Y	N	Y	Y	Y	N	NR	NA	Y	N	N	-	-	Fair

Y. yes; N, no; CD, cannot determine; NA, not applicable; NR, not reported.

**Table 2:** Demographics.

Study	Study Design	Women Included	Age, Mean	Operative Technique	Number of Myomas	Pregnancies	Pregnancy Outcomes		Preterm	Mode of Delivery	
							Live Births	Miscarriages		C	V
Myo Sun Kim [6]	Retrospective cohort study	340	34.3 ± 2.9	LSM	2.0 ± 1.6	54	44	3	0	50	3
Stéphanie Huberlant [7]	Retrospective cohort study	53	35.4 + 5.3	RALSM	2 ± 1.57	28	22	4	1	17	7
Tina Sybille Bernardi [8]	Retrospective cohort study	65	33	LSM	2 (median)	55	38	13	3	26	21
Yu-Jin Koo [9]	Retrospective cohort study	523	-	LSM	Single myoma 339 Multiple myomas 184	523	401	68	54	350	100
Prapas [10]	Prospective cohort study	273	34.9 ± 5.2	LSM/LAIM (Laparoscopic assisted intracapsular myoma)	2.4 ± 0.7	148	137	8	NA	127	10
Paul P. G. [11]	Retrospective cohort study	182	32.4 ± 5.2	LSM	2 (median)	94	79	NA	8	63	16
Pepin [12]	Retrospective cohort study	101	37 ± 6	LSM	3.5 ± 3.4	110	60	34	8	54	6
Yu-cui Tian [13]	Retrospective cohort study	179	30.28 ± 3.99	LSM	NA	82	61	9	3	20	41
Alessandro Arena [14]	Retrospective cohort study	164	36.0 + 4.4	LSM	2.9 ± 2.4	103	70	29	8	57	13
Ordás [15]	Retrospective cohort study	112	35.91 ± 5.517	LSM	1.8 ± 1.5	36	31	4	NA	16	15
Guangping Wu [16]	Retrospective cohort study	224	32.4	LSM	3.9	253	173	14	9	95	63
Kumakiri [17]	Retrospective cohort study	1334	33.6 + 3.4	LSM	3.5 ± 3.6	221	111	47	NA	29	82
Fukuda [18]	Retrospective cohort study	48	35.3 ± 3.4	LSM	2.6 ± 2.2	48	NA	NA	7	34	14
Rebecca Flyckt [19]	Retrospective cohort study	10	33.5 ± 4.4	LSM	NA	9	9	4	NA	NA	NA
		15	34 ± 3.8	RALSM		5	5	0			
Pitter [20]	Retrospective cohort study	426	37.9 ± 5.8	RALSM	NA	114	NA	37	11	NA	NA
Cela [21]	Retrospective cohort study	48	35.2 ± 6.0	RALSM	1 (median)	7	6	0	0	5	2
Yeon Hee Hong [22]	Retrospective cohort study	56	40.6 ± 6.6	SPLSM	2.3 ± 2.2	42	39	2	3	36	3
Ji Ye Kim [23]	Prospective case–control study	135	-	LSM	NA	12	7	3	NA	4	3
				SPLSM		9	6	1		6	0

LSM. Laparoscopic myomectmy; RALSM, Robotic assisted laparoscopic myomectomy; SPLSM, Single port laparoscopic myomectomy; LAIM, Laparoscopic assisted intracapsular myoma.

**Table 3:** Types of myoma and fertility outcomes.

Study	Number of Women	Number of Pregnant Women	Location of Myoma	Number of Myomas, Mean
Tina Sybille Bernardi [8]	65	44	Partially intramural—51	2
			Intramural—25	
			Intramural with endometrium contact—14	
			Submucous—9	
			Subserous fibroids—33	
			Interligamentary—6	
			Pedunculated—2	
Pepin [12]	101	76	Submucosal—13	2
			Intramural—67	
			Subserosal—48	
Guangping Wu [16]	224	253	Subserosal—31	3.9
			Intramural—169	
			Submucosal—7	
			Intramural and submucosal—17	
Paul P. G. [11]	182	93	Submucosal—27	2
			Intramural—179	
			Subserosal—68	
			Cervical—14	

**Table 4:** Intraoperative suture type and number of uterine ruptures.

Study	Study Design	Intraoperative Technique	Number of Uterine Ruptures	Number of Women
Myo Sun Kim [7]	Retrospective cohort study	Multilayer suture	2	415
Fukuda [18]	Retrospective cohort study	Multilayer suture	0	48
Tina Sybille Bernardi [8]	Retrospective cohort study	Intracorporeal	2	65
Yu-Jin Koo [9]	Retrospective cohort study	Intracorporeal	3	523
Alessandro Arena [14]	Retrospective cohort study	Barbed	0	83
		Non-barbed	0	81
Paul P. G. [11]	Retrospective cohort study	Barbed	0	115
		Non-barbed	0	120

**Table 5:** Number of myomas and fertility outcomes.

Study	Age (Mean)	Number of Myomas	Number of Pregnancies	Pregnancy Outcomes		Preterm	Mode of Delivery	
				Live Births	Miscarriages		C	V
Myo Sun Kim [6]	34.3 ± 2.9	2.0 ± 1.6	54	44	3	0	50	3
Prapas [10]	34.9 ± 5.2	2.4 ± 0.7	148	137	8	NA	127	10
Pepin [12]	37 ± 6	3.5 ± 3.4	110	60	34	8	54	6
Alessandro Arena [14]	36.0 + 4.4	2.9 ± 2.4	103	70	29	8	57	13
Ordás [15]	35.91 ± 5.517	1.8 ± 1.5	36	31	4	NA	16	15
Guangping Wu [16]	32.4	3.9	25	173	14	9	95	63
Kumakiri [17]	33.6 + 3.4	3.5 ± 3.6	221	111	47	NA	29	82

C means cesarean delivery; V means vaginal delivery.

## Data Availability

Not applicable.
